# A Role for the Adult Fat Body in *Drosophila* Male Courtship Behavior

**DOI:** 10.1371/journal.pgen.0030016

**Published:** 2007-01-26

**Authors:** Anna A Lazareva, Gregg Roman, William Mattox, Paul E Hardin, Brigitte Dauwalder

**Affiliations:** 1 Department of Biology and Biochemistry, University of Houston, Houston, Texas, United States of America; 2 Department of Molecular Genetics, University of Texas, M. D. Anderson Cancer Center, Houston, Texas, United States of America; 3 Department of Biology, Texas A&M University, College Station, Texas, United States of America; Princeton University, United States of America

## Abstract

Mating behavior in *Drosophila* depends critically on the sexual identity of specific regions in the brain, but several studies have identified courtship genes that express products only outside the nervous system. Although these genes are each active in a variety of non-neuronal cell types, they are all prominently expressed in the adult fat body, suggesting an important role for this tissue in behavior. To test its role in male courtship, fat body was feminized using the highly specific Larval serum protein promoter. We report here that the specific feminization of this tissue strongly reduces the competence of males to perform courtship. This effect is limited to the fat body of sexually mature adults as the feminization of larval fat body that normally persists in young adults does not affect mating. We propose that feminization of fat body affects the synthesis of male-specific secreted circulating proteins that influence the central nervous system. In support of this idea, we demonstrate that Takeout, a protein known to influence mating, is present in the hemolymph of adult males but not females and acts as a secreted protein.

## Author Summary

Many animal species have developed elaborate courtship rituals. Male courtship behavior in fruit flies, *Drosophila melanogaster,* consists of a well-defined series of behaviors. Specific regions of the male brain are required for successful courtship, and these neuronal cells require expression of male-specific proteins for normal function. We show here that male factors produced by a secretory tissue that surrounds the brain are also required for successful courtship. When these factors were eliminated, the frequency of several aspects of courtship was reduced, leading to fewer successful matings. At least one of these factors is secreted into the circulatory system of the fly and acts as a secreted protein. Male courtship may therefore require the interaction of endocrine factors with specific parts of the nervous system.

## Introduction

Male courtship in Drosophila melanogaster consists of an innate sequence of behavioral steps that are well characterized [1,2]. The sex-specific ability to perform this behavior is controlled by the same master regulatory genes that control somatic sex differentiation in general *(Sex-lethal [Sxl], transformer [tra],* and *transformer-2 [tra-2]).* Tra and Tra-2 are splicing factors that directly control the sex-specific alternative splicing of *doublesex (dsx)* and *fruitless (fru)* mRNAs, resulting in the formation of dsxM, dsxF, and fruM proteins. While *dsx* primarily controls sexual differentiation outside of the central nervous system (CNS) [3,4], *fru* is a key regulator of male courtship behavior [2,5–8]. Severe *fru* mutations abolish courtship [6], while some less severe loss-of-function mutations result in male–male courtship. Male-specific FRU protein (FRU-M) is expressed in numerous cells in the nervous system, including the regions that have previously been identified as important for male courtship behavior [2,5–7,9–14]. It has recently been demonstrated that expression of FRU-M in these cells in females is sufficient to induce courtship behavior toward normal females, demonstrating the prominent role of *fru* in regulating sex-specific behavior [15–17]. However, the level of courtship in these females is reduced in comparison to wild-type males, suggesting that other components are also required that have not been transformed by the expression of FRU-M.

Although the importance of sexual identity in the CNS is well established, recent studies on genes affecting courtship suggest that other tissues also play required roles. Two of the best studied among these genes, *takeout* and *Gr68a,* are expressed male specifically and are involved in male courtship behavior. *Gr68a* belongs to a family of G protein–coupled receptors and is expressed very specifically in the chemosensory neurons of a few male-specific taste bristles in the foreleg [18]. Removal of *Gr68a* by RNA interference affects courtship behavior, suggesting that it may play a role as a receptor in the recognition of female pheromones by the male [18].

The Takeout protein is preferentially expressed in male heads [19]. It is similar to small carriers of lipophiles from both insects and mammals, particularly the Juvenile Hormone–binding proteins from other insects. Mutant males show reduced courtship behavior, and a mutation in *takeout* interacts genetically with *fru* to affect courtship. Non–sex-specific expression of *takeout* is found in the antennae, the main olfactory and auditory organ in flies and (under starvation conditions) in the cardia. Sex-specific expression is observed in the fat body surrounding the brain. No expression was observed in the brain [19]. When the *takeout* promoter was used to feminize tissues in males by forced expression of the female-specific transformer protein (TraF), male courtship behavior was drastically reduced [19]. Importantly, the *takeout-Gal4* driver that was used in these experiments is expressed in all *takeout*-expressing tissues. It is therefore unclear which of these tissues is crucial for courtship.

The fat body, which also expresses a number of male-specific proteins in addition to *takeout,* is a candidate for mediating the observed behavioral effect [20]. The fat body is a major secretory tissue in both larvae and adults. It secretes factors into the hemolymph, the fly's circulatory system. A sex-specific role for the fat body has so far been documented only in females, where, under the control of the sex determination pathway, it produces large amounts of secreted yolk proteins [21]. Here we examine whether male sexual identity in the fat body is required for normal courtship behavior.

## Results

### Generation of Gal4 Driver Lines Specific to Larval and Adult Fat Body

**Figure 1:**
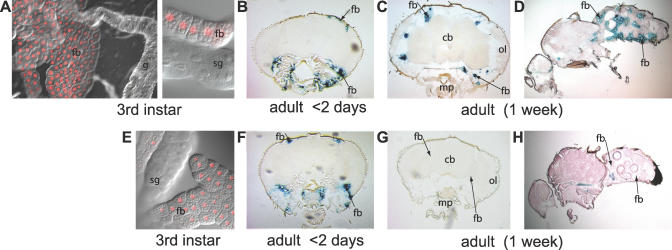
Stage-Specific Fat Body Drivers Fat body (fb)-specific Gal4 activity driven by the 3.1-kb *Lsp2* promoter fragment (A–D) or the 0.68-kb *Lsp2* promoter fragment (E–H) was detected in larvae and in sections from adult heads and bodies using a *UAS-dsRed* (A and E) or a *UAS-lacZ* reporter gene (B–D and F–H). Staining in larvae and flies younger than 2 d [representing larval fat body staining (A, B, E, and F)] and in 1-wk-old flies [adult fat body staining (C, D, G, and H)] is shown. For reference, the positions of the central brain (cb), optical layers of the brain (ol), mouthparts (mp), gut (g), salivary glands (sg), and fat body (fb) are indicated.

To test whether feminization of the fat body is sufficient to disturb male courtship behavior, it was first necessary to generate a promoter that could be used to drive TraF expression in a manner that is both highly localized to this tissue and non–sex specific (to avoid artificial feedback regulation). An added complexity of these experiments is that there are two physiologically distinct types of fat body found in the adult. Fat body that originates in larval stages (larval fat body) persists into the first few days of adult life, when it is replaced by adult fat body [22]. To address the possibility that larval and/or adult fat body might play a role in courtship, we chose the well-studied promoter of the *Larval serum protein 2 (Lsp2)* gene for this purpose. The *Lsp2* gene has been shown to be specifically expressed in the larval and adult fat body, including the fat body surrounding the adult brain where *takeout* is expressed [23,24]. We used a 0.68-kb and a 3.1-kb promoter *Lsp2* fragment that have both been shown previously to direct fat body–specific expression when fused to a *lacZ* reporter [25]. We generated transgenic flies in which these fragments were each fused to the yeast Gal4 coding region. Gal4 expression in the resulting *Lsp2*-*Gal4* flies was visualized by crossing them to *UAS-dsRed (Stinger)* flies or to *UAS-lacZ*–carrying flies. We found that the 3.1-kb fragment supports expression in both larval and adult fat body (Figure 1A through 1D). Expression from the 0.68-kb fragment is limited to the larval fat body (Figure 1E through 1H), which is present in larvae and in young, but not older, flies. Staining in both heads and bodies was specific to fat body; no staining was observed in the brain, the antennae, the maxillary palps, or the mouth parts even after extended incubation (unpublished data). We conclude that our *Lsp2-Gal4* transgenes are specifically expressed in fat body cells and therefore well suited to examine the specific role of fat body in courtship.

### Feminization of the Adult, but Not the Larval, Fat Body Affects Male Courtship Behavior

**Figure 2:**
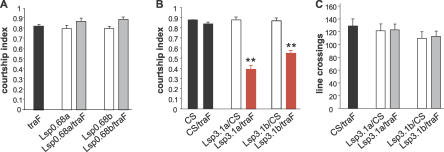
Feminization of the Adult, but Not the Larval, Fat Body Affects Male Courtship (A and B) CIs of various males toward *Canton-S* virgin females. Males carrying the *UAS-traF* (black bar) or the *Lsp2-Gal4* (white bars) transgenes individually are compared to *0.68Lsp2-Gal4/UAS-traF* males (A, gray bars) and *3.1Lsp2-Gal4/UAStraF* males (B, red bars). Courtship was reduced only in *3.1Lsp2-Gal4/UAS-traF* males. The results from two different transgenic *Lsp2-Gal4* lines (designated a and b) are shown for each transgene (*n* = 10 for each group; **indicates indices that were significantly different from those of parental strains, *p* < 0.001). Performance in a short-term activity assay (number of line crossings) is shown for *3.1Lsp2-Gal4/UAS-traF* males and the corresponding parental lines (C) (*n* = 10 for each group). No differences between genotypes were observed.

To examine the role of the fat body in male courtship, *3.1Lsp2-Gal4* and *0.68Lsp2-Gal4* flies were next crossed to *UAS-TraF* flies. Expression of TraF sexually transforms male cells into female cells [26]. The resulting *3.1Lsp2-Gal4/UAS-TraF* and *0.68Lsp2-Gal4/UAS-TraF* males with feminized fat bodies were tested for their ability to court wild-type females in a standard courtship assay [7,27]. Two independent transgene insertions were tested for each construct. The results are shown in Figure 2, represented as the courtship index (CI), which is a measure of the time that a male spends performing any of the steps of courtship during a fixed observation period. XY flies of the genotype *0.68Lsp2-Gal4/UAS-TraF* courted normally (Figure 2A), indicating that male identity in larval fat body is not required. In contrast, a significant reduction in courtship was observed in *3.1Lsp2-Gal4/UAS-TraF* males in comparison to control flies (Figure 2B). To characterize the observed reduction in courtship in more detail, we examined several individual courtship steps (Table 1). The latency to initiate courtship (i.e., the first orientation toward the female) is not affected in the mutants. In contrast, the relative frequency of wing extensions and attempted copulations is reduced. This may be related to the fact that courtship is poorly sustained and often interrupted in the mutants. It has been suggested that later steps of courtship require a previous “buildup phase” in the earlier steps. The number of attempted copulations is also low in *Lsp0.68a* flies but equally so in the control and the TraF combination for unknown reasons. In summary, these results show that feminization of adult fat body severely lowers the probability that a male will sustain courtship beyond the first steps, although all steps, including copulation, can be carried out. To determine whether the effect seen in *3.1Lsp2-Gal4/UAS-TraF* flies might be explained by general sluggishness, we carried out short-term activity assays to measure the movement of males in the same chambers used for courtship assays but without a female present [28]. The activity of the courtship-defective *3.1Lsp2-Gal4/UAS-TraF* flies was equal to that of the control flies (Figure 2C). From these data, we conclude that specific feminization of the adult fat body is capable of disturbing male courtship. In contrast, feminization of the larval fat body, which is present through metamorphosis into early adulthood, has no effect on courtship. These results show that the adult fat body makes an important and unique contribution to male courtship behavior.

**Table 1:**
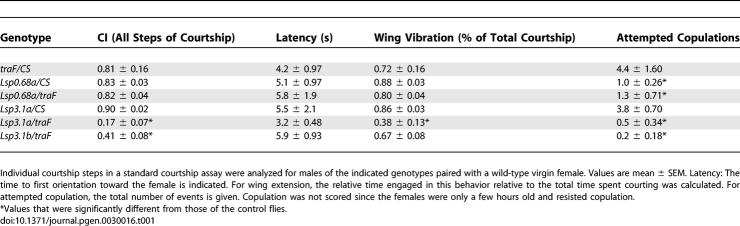
Courtship Elements in Males with Feminized Fat Bodies

### Masculinization of the Fat Body Enhances Courtship in *Fru^M^* Females

**Figure 3:**
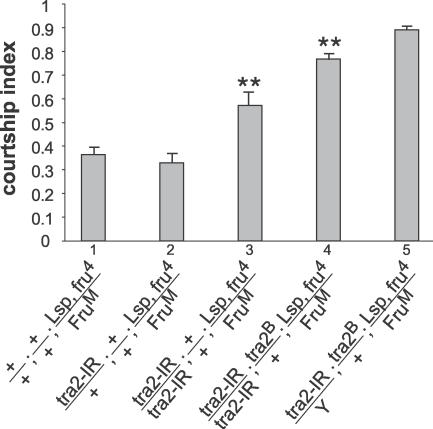
Masculinization of the Fat Body Enhances the Courtship of *Fru^M^* Females CIs of various females toward *Canton-S* virgin females are shown. All animals were *Fru^M^*, carried the *Lsp3.1-Gal4* (Lsp) fat body driver on a *fru^4^* chromosome, and were female (except for column 5). The presence of two copies of *tra2-IR* significantly enhanced courtship, which was further increased when the females were heterozygous for *tra-2^B^/+* at the same time. Column 5: Control male siblings of the females in column 4. Animals were kept at 29 °C following eclosion until testing which was done at room temperature (*n* = 10 for each group; **indicates indices that were significantly different from those of *Fru^M^* females [column 1], *p* < 0.05). The CI of *tra2-IR/tra2-IR; tra-2^B^/+; Lsp, fru^4/^Fru^M^* females is statistically not different from that of the control males.

It has recently been shown that expression of FRU-M in *fru-*expressing circuits in females is sufficient to induce their courtship toward wild-type females [15–17]. However, while induction of FRU-M in these cells causes females to display male courtship behavior, their overall CI is reduced in comparison to wild-type males. One likely explanation for this is that male identity of additional cells other than those expressing FRU-M is required for normal levels of male courtship. For example, courtship in *Fru^M^* females may be low because Takeout and other male-specific fat body factors are absent. To test whether fat body masculinization can enhance the courtship of *Fru^M^* females, we used the *Lsp3.1-Gal4* fat body driver to express a *tra-2* interfering RNAi construct *(UAS-tra2-IR)* [29] specifically in fat body cells of *Fru^M^* animals and tested their courtship (Figure 3). Mutations in *tra-2* lead to the transformation of chromosomal females into phenotypic males with male courtship behavior [2,3,30], and expression of *tra-2-IR* has previously been shown to partially masculinize the cells in which it is expressed in a dosage- and temperature-dependent manner [29]. *Fru^M^* females carrying the *Lsp3.1-Gal4* driver but not the *UAS-tra2-IR* responder courted (Figure 3, column 1) with a CI of 0.37 ± 0.03. One copy of the *UAS-tra2-IR* responder did not affect courtship, but the introduction of two copies of *UAS-tra2-IR* significantly increased the courtship of *Fru^M^* females, to 0.57 ± 0.06 (Figure 3, columns 2 and 3). These results indicate that male-specific factors from the fat body enhance the male behaviors displayed by females with a male-specific *fru* neuronal circuit and confirm the important role of the fat body in promoting courtship. Because it is likely that the knockdown of Tra2 function in the above genotype by *UAS-tra2-IR* is incomplete, we also tested its ability to enhance courtship in flies with reduced endogenous *tra-2* levels. For this purpose, females heterozygous for a *tra-2* loss-of-function mutation were used. Previous studies have shown that such heterozygotes do not perform any aspects of male courtship behavior [2]. As expected, reduced endogenous *tra-2* function allowed the *UAS-tra2-IR* transgene to increase courtship to a still higher level (to 0.77 ± 0.03) in *Fru^M^* females (column 4).

### Sex-Specific *fru* Expression Is Independent of the Sexual Identity in the Fat Body

**Figure 4:**
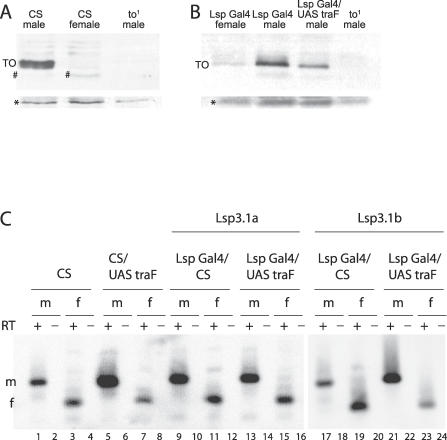
Sex-Specific *fru* Expression Is Independent of the Sexual Identity in the Fat Body (A and B) Takeout protein levels are reduced in males with feminized fat body. Western blot analysis of head extracts is shown. Equal amounts of protein were loaded in each lane. Antibodies raised against the carboxyl-terminal region of the Takeout protein recognize a protein of the expected size for Takeout (27 kDa) in males but not in females and *to^1^* mutant males (A) (#: background band). Takeout levels are reduced in *Lsp-Gal4/UAS-traF* males compared to males heterozygous for the *Lsp-Gal4* transgene (B). A background band (*) is shown to visualize comparable protein amounts in all lanes. (C) Alternative splicing of *fru* pre-mRNA. RT-PCR was performed on heads from males and females using primers that specifically amplify male and female-specific *fru* mRNAs. PCR products were visualized by Southern blot hybridization with sex-specific *fru* probes. The genotypes of the flies are indicated. *Canton-S (CS)* flies are shown as a control. Transgenic *3.1Lsp2-Gal4* lines were crossed to either *CS* (control flies) or *UAS-traF* flies. RT–PCR reactions were performed in either the presence (+) or absence (–) of reverse transcriptase (RT). Two independent transgenic *3.1Lsp2-Gal4* lines were examined (lines a and b).

When and how does the adult fat body interact with the CNS to affect courtship? Sexual identity in the brain is established by late pupal development [31,32]. It is therefore unlikely that feminization of the adult fat body affects courtship by influencing the sexual identity of the brain itself. However, it is possible that signals from the fat body are required to maintain male identity and function within the CNS. We therefore examined *fru* expression in males with feminized fat body. *fru* is a major regulator of sexual identity in the brain, and normal male courtship requires male-specific *fru* transcripts and the resulting male-specific FRU-M protein [5,6]. FRU-M is also required to prevent cell death in a cluster of sexually dimorphic cells in the brain which are important for courtship [8]. In females, *fru* RNA is spliced differently and no FRU-M is expressed [33]. If feminization of adult fat body in males affected a regulatory input upstream of *fru*, we would expect to observe *fru* transcripts spliced in a female mode in these animals. We first verified feminization of the fat body by examining the protein levels of Takeout since it is preferentially expressed in male head fat body. The antibody specifically recognizes a protein of approximately 27 kDa, the expected size of the Takeout protein (Figure 4A) in heads of males. As expected, Takeout protein levels were reduced in *3.1Lsp2-Gal4/UAS-traF* males with feminized fat bodies (Figure 4B). We then examined the *fru* splicing pattern in heads of adult *3.1Lsp2-Gal4/UAS-traF* flies by reverse transcription–PCR (Figure 4C). For comparison, *fru* expression in the parental lines is shown. Two independent transgenic lines were examined. Feminization of the fat body did not alter *fru* splicing in these animals as shown by the presence of the correct sex-specific splice products in males and females (lanes 13 and 15; 21 and 23). We conclude from these results that sex-specific splicing of *fru* pre-mRNA occurs in the brain independently of sexual identity in the surrounding fat body. Because unintended expression of TraF within the CNS would also be expected to alter *fru* expression, these results further support the above observation that the *Lsp-Gal4* driver lines are not active within the CNS.

### The Male-Specific Takeout Protein Is Secreted from the Fat Body into the Hemolymph and Acts as a Secreted Protein

**Figure 5:**
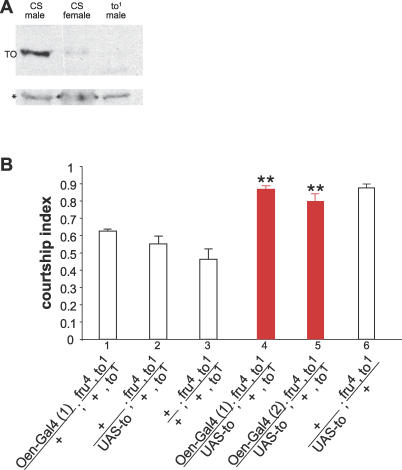
Takeout Is Present in the Hemolymph and Is Functional when Expressed from Oenocytes (A) Takeout is present in the hemolymph. Western blot of isolated hemolymph from wild-type (*Canton-S [CS]*) males and females and of *to^1^* mutant males probed with anti-Takeout antiserum as in Figure 4A. Hemolymph from an equal number of animals was loaded in each lane. An approximately 27-kDa protein was recognized in hemolymph from male flies, but not from females and *to^1^* mutant males. A background band (*) is shown to visualize comparable protein amounts in all lanes. (B) Takeout expression in oenocytes rescues the *takeout* courtship defect. CIs of various males towards *Canton-S* virgin females are shown. Males carrying the *UAS-takeout (UAS-to)* or the *Oenocyte-Gal4 (Oen-Gal4)* transgenes individually in a *fru^4^, to^1^/+, to^1^* background (columns 1 and 2) are compared to *Oen-Gal4/UAS-to* males in the same genetic background (columns 4 and 5). Results for two different *Oen-Gal4* drivers are shown (red bars, columns 4 and 5) (*n* = 10 for each group; ** indicates indices that were significantly different from those of mutant *fru^4^, to^1^/+, to^1^* males [column 3], *p* < 0.01). Column 6: Control males. All males had wild-type eye color. The better performance of males with the *Oen-Gal4* driver only (compared to just the mutant alone) is likely due to background effects.

Our results suggest that the fat body and the brain interact in the adult male to allow efficient courtship. We hypothesize that the fat body produces male-specific proteins that are secreted into the hemolymph, where they can access and influence brain functions downstream of male-specific *fru* expression. Although multiple proteins may be required for this interaction, Takeout is the only male-specific protein from fat body known to be required in courtship. Moreover, this protein contains a putative signal peptide, suggesting that it is secreted [19]. We therefore tested whether Takeout is present in the circulating hemolymph. Western blots on hemolymph revealed that Takeout protein circulates in males, but not in females or *takeout^1^ (to^1^)* mutant males (Figure 5A). Thus, at least one protein required for optimal mating is present in the hemolymph of males, consistent with the idea that fat body affects behavior through diffusible factors. If Takeout affects courtship as a circulating protein, one would predict that it could do so also if secreted into the hemolymph from cells other than fat body cells. Oenocytes are subcuticular abdominal cells with multiple endocrine functions, among them the production of cuticular hydrocarbons [26]. We used oenocyte-specific Gal4 drivers [26] to express Takeout in these cells and examined whether expression of Takeout in oenocytes was capable of rescuing the courtship defects of *takeout* mutants. We have previously shown that courtship in males homozygous for the *to^1^* mutation and heterozygous for a mutation in *fru* is reduced by about one third and that this defect is rescued by supplying *takeout* from a genomic rescue construct [19]. Using two different oenocyte-specific Gal4 driver lines (lines 72 and 1407), we expressed Takeout in *fru^4^/+, to^1^/to^1^* mutant males and examined their courtship (Figure 5). Full rescue of the mutant courtship phenotype was observed with both drivers. These results suggest that Takeout functions in courtship as a secreted protein. They also argue that Takeout does not play a developmental role but rather has a physiological role in adults.

## Discussion

### A Role for a Non-neuronal Tissue in Courtship Behavior

It is well established that the courtship ritual of *Drosophila* males depends on the male identity of the nervous system. [10,26,34–38]. Male-specific expression of FRU-M was found in the regions of the nervous system that have been implicated in the regulation of male courtship, and it has recently been demonstrated that the neuronal network defined by FRU-expressing cells is necessary and sufficient for male mating behavior [16,17]. However, while induction of FRU-M in these cells causes females to display male courtship behavior, their overall CI is reduced in comparison to wild-type males. We show here that male differentiation of fat body is required in addition to male-specific circuits in the CNS for normal courtship. We therefore suggest that after male sexual differentiation and the competence to support male courtship are established in the nervous system, additional secreted factors produced by fat body cells are required to support and promote normal courtship behavior. This is reminiscent of vertebrates where sexual differentiation and behavior are regulated by hormones and opens the possibility that, in flies, diffusible factors play a similar role.

Dsx and Fru are thought to function in two alternate and parallel branches of the sex determination regulatory pathway that are both controlled by TraF together with Tra-2. A mutation in *dsx* strongly affects expression of several known sex-specific fat body genes [19,20,39], directly indicating a prominent role of the *dsx* branch of the pathway in fat body sexual differentiation. The contribution of fat body to courtship behavior described here is consistent with previous studies indicating that *dsx* has an important role in male courtship behavior. For instance, *dsx* mutants have reduced courtship that parallels that seen in flies with TraF feminized fat body. The basic elements of courtship are in each case displayed but with lower probability [40]. A recent study by Shirangi et al. [41] demonstrated that both *fruM* and *dsx* (together with non–sex-specific *retained [retn]*) affect both male and female behaviors and that *dsxM* is needed in addition to *fruM* for efficient male courtship. The cells and tissues in which *dsxM* is required for this function were not defined. Based on our results, we propose that the fat body is one of them. Reduction of *tra-2* in the fat body of females *(*by *tra2-IR)* leads to the generation of *dsxM*, since *dsxM* and *dsxF* are generated by sex-specific alternative splicing controlled by *tra-2* (together with *tra*). DsxM then activates *dsxM*-dependent transcription of male-specific genes such as *takeout* and other genes. Interestingly, we have previously found that expression of *takeout* itself is affected by both *dsx* and *fru* mutations [19]. While we found significant enhancement of courtship in *Fru^M^* females by expressing two copies of *tra2-IR* in the fat body, courtship improved even more when the animals were heterozygous for *tra-2* in addition. Heterozygosity for *dsx* and expression of dsxM in females by themselves do not lead to male courtship behavior [2,41]. However, in combination with Fru-M, courtship is enhanced, as shown by Shirangi et al. [41] and our present results. Whether this effect results purely from a further reduction in Tra2 levels and masculinization within the fat body or reflects effects of reduced Tra2 on other tissues remains to be determined.

The finding that feminization of the fat body in adult flies affects courtship may seem to contradict previous findings that male sexual behavior is determined during pupal development and not reversible in adults [32]. However, the previous studies quantified how many of the steps in the courtship ritual were present but did not quantify how often they were performed. Males with feminized fat body can perform all steps of courtship, but they display them less often. Thus, they would have been scored as unaffected in the previous studies.

### Diffusible Products May Play a Role in Courtship Behavior

In addition to the multiple roles of diffusible neuropeptides in growth, nutrition, molting, and female reproduction [42], there is evidence for diffusible molecules with sex-specific function. Excitatory and inhibitory pheromones play an important part in courtship behavior [43,44]. There is also evidence that diffusible factors can influence courtship behavior. Several proteins in male seminal fluid have been shown to enter the female hemolymph and induce changes in oviposition as well as receptivity to subsequent courting males [45–49]. In addition, the courtship defects seen in the *dsf* mutant which affects a putative steroid receptor may indicate a role for diffusible factors [50,51]. Belgacem and Martin [52] have shown that Juvenile Hormone affects sexually dimorphic locomotion rhythms in *Drosophila*. Since the fat body is a major secretory tissue, it is likely that it exerts its function on the brain through molecules secreted into the hemolymph, the fly's circulatory system. In support of this hypothesis, we find that Takeout is present in the hemolymph of males and acts as a secreted factor. Takeout therefore appears to be one of the diffusible factors that mediate the communication between the fat body and the brain that allows for efficient courtship. However, the effect of a mutation in *takeout* alone on CI is significantly lower than the pronounced effect of feminizing all adult fat body. This indicates the presence of other factor(s) produced by this tissue with a strong role in courtship, possibly including other members of the *takeout* gene family. The presence of a number of such factors might allow the regulation of a male's propensity to engage in male courtship behavior in response to environmental factors (i.e., age, nutritional status). In this regard, it is interesting that *takeout* expression is induced in a variety of tissues in response to starvation [53].

### Sexual Maturation in Male Flies

It has been demonstrated that behavioral competence for a number of behaviors, including general activity, the response to stimuli, and courtship behavior, is reduced in young flies, and it has been suggested that the CNS continues to differentiate after eclosion [54]. Likewise, the adult pheromone profile develops in the newly formed cuticle during the first few days of adult life [55,56]. Therefore, the sex-specific functions of the brain and the fat body may become realized only once a general competence for complex behaviors is reached. Freshly eclosed males start efficient courtship after 2 to 3 d [54,57], which coincides with the appearance of adult fat body. Interestingly, in *apterous* mutant flies in which larval fat cells persist longer and the appearance of adult fat body is delayed [58], mature male courtship develops slower and is reduced in its intensity [59]. Given our results that adult fat body is required for efficient courtship, it is conceivable that adult fat body not only is required for mature male courtship but also plays a role in the maturation of this behavior.

## Materials and Methods

### Fly strains.

Flies were kept on standard cornmeal/sugar–based food at room temperature under noncontrolled light conditions. The *to^1^* mutation is present in Bloomington stock 2541 (*sn^w^; ry^506^, to^1^;* [19]). The strains *w; UAS-traF* (stock number 4590) and *w^1118^; P{UAS-RedStinger}4/CyO* (stock number 8546) were obtained from the Bloomington Drosophila Stock Center at Indiana University (http://flybase.bio.indiana.edu). *Oenocyte-Gal4 1407/CyO* was a gift from Jean-François Ferveur (Centre National de la Recherche Scientifique, Dijon, France), *Fru^M^* was a gift from Barry Dickson (Research Institute of Molecular Pathology, Vienna, Austria), and *Tra2-IR* was a gift from John Belote (Syracuse University, Syracuse, New York, United States).

### Generation of transgenes.

*0.68Lsp2-Gal4* and *3.1Lsp2-Gal4* transgenic flies were made by PCR amplification of 0.68 kb and 3.1 kb, respectively, of the *Lsp2* promoter region from *Canton-S* genomic DNA, followed by cloning into the NotI site of *pCaSpeR4-GATN*. The construct was verified by sequencing. pCaSpeR4-GATN was made by subcloning the Gal4 coding region of *pGATN* [60] as a KpnI/SpeI fragment into *pCaSpeR4* [61]. Transgenic lines were established in a *w^1118^* background.

The primers used were (NotI linker sequences are given in italics) 5′-*ATAAGAATGCGGCCGC*GGCGAACAAAGCAAACTTATCAAGG-3′, 5′-*ATAAGAATGCGGCCGC*GTTCACCCACATGTGTCTTGCCGA-3′, and 5′-*ATAGTTTAGCGGCCGC*CTCGAGTCGATTCGAATCGTAACG-3′.

*UAS-to* flies were generated by PCR amplification of the *takeout* open reading frame from reverse-transcribed RNA of *Canton-S* flies and subsequent cloning into the pUAST vector. The construct was verified by sequencing. Transgenic flies were established in a *w^1118^* background.

### Sections and stainings.

For X-gal staining, 10-μm frozen sections were cut on a Leitz cryostat, dried at room temperature for 5 min, fixed in 1% glutaraldehyde in PBS for 10 min, and washed in PBS. X-gal staining was done as described [62]. For dsRed detection, unfixed larvae were dissected and imaged in PBS, 50% glycerol on a Zeiss LSM 510 confocal microscope.

### Courtship assay and short-term activity assay.

Assays were performed as described [19]. Tested males were 4 to 6 d old.

### RNA preparation and RT-PCRs.

RNA was prepared using the *Totally RNA* kit (Ambion, http://www.ambion.com). DNase-treated RNA was reverse transcribed using the Superscript II kit (GIBCO, http://www.invitrogen.com) and *fru* RT-PCR was performed as described previously [63].

### Anti-Takeout antibody generation and affinity purification.

Takeout sequences coding for the 67 carboxyl-terminal amino acids were amplified by PCR and cloned into the pGEX-6P1 expression vector (Amersham Biosciences, http://www.amersham.com) as an EcoRI fragment. This region has only limited similarity to other *takeout* family members. The primers used were (linker sequences given in italics): 5′-*CGGAATTC*TCGGAGGCCATTTACAAGGAG-3′ and 5′-*GCGAATTC*TCAAAACTTAGATCTGTCT-3′. GST-tagged Takeout protein was expressed in BL21 cells and purified over a glutathione Sepharose 4B column as described by the supplier, except that 2% Sarkosyl was added to solubilize the protein. Polyclonal antibodies were made in rabbit. For affinity purification, rabbit IgG was purified from serum using Econo-Pac Serum IgG purification columns (Bio-Rad, http://www.bio-rad.com) as described by the supplier. Takeout peptide was coupled to Affi-Gel 10 matrix (Bio-Rad) in 0.1 M MOPS (pH 7.5), 2% Sarkosyl. Unreacted groups were blocked with 0.1 M ethanolamine at the end of the coupling reaction. The column was washed with 0.1 M glycine followed by washes with 100 mM Tris-HCl (pH 6.8). The purified IgG fraction, diluted in 100 mM Tris-HCl (pH 6.8), was applied to the column and eluted with 0.1 M glycine (pH 2.4). Fractions were neutralized by the addition of 0.1 volume of 1 M Tris-HCl (pH 8.8) and concentrated using a Centricon 10 unit. Glycerol was added to a final concentration of 10%, and aliquots were kept frozen at −80 ° C.

### Western blots.

Preparation of extracts and Western blotting were performed as described [64]. To visualize Takeout protein in heads, 2 OD_595_ of total protein extract was loaded in each lane. Hemolymph isolated from equal numbers of animals (between 50 and 100) was loaded per lane to visualize Takeout in hemolymph. For protein extracts, animals were entrained in a 12 h/12 h light/dark cycle, and protein/hemolymph was collected at ZT21. Anti-Takeout was used at a dilution of 1:2,000. HRP-coupled anti-rabbit (Sigma, http://www.sigmaaldrich.com) was used as secondary antibody at a dilution of 1:1,000.

### Hemolymph isolation.

We followed a protocol kindly provided by Ravi Ram Kristipati and Mariana Wolfner, Cornell University, Ithaca, New York, United States [46]. From 30 to 50 flies were punctured in the thorax with a fine Tungsten needle and placed head down into an Eppendorf tube that was punctured with a 25-gauge needle and plugged with cotton. The tube was inserted into another Eppendorf tube and centrifuged at 5,500 rpm for 5 min. The tiny droplet at the bottom was collected and quick-frozen on dry ice.

### Statistics.

Analyses were performed with JMP 2.0 statistical software (SAS Institute, Inc., http://www.sas.com). Following initial analysis of variance, comparisons between groups were by unplanned multiple comparisons using Tukey-Kramer analysis at α = 0.05.

## Abbreviations

CI: courtship index
CNS: central nervous system
*dsx*: *doublesex*
*fru*: *fruitless*
FRU-M: male-specific FRU protein
*Lsp2*: *Larval serum protein 2*
*Sxl*: *Sex-lethal*
*to*^*1*^: *takeout* ^*1*^
*tra*: *transformer*
*tra-2*: *transformer-2*
TraF: female-specific transformer protein
